# The regulatory role of circGDI2 in hepatocellular carcinoma proliferation and glycolysis with the involvement of m6A modification

**DOI:** 10.1016/j.ncrna.2025.11.006

**Published:** 2025-12-04

**Authors:** Shiyi Chen, Hongxiang Xia, Shuwei Chen

**Affiliations:** a Department of Hepatobiliary Surgery, The Affiliated Nanhua Hospital, Hengyang Medical School, University of South China, No. 336, Dongfeng South Road, Zhuhui District, 421001, Hengyang City, Hunan Province, China; b Interventional Diagnostic Center, The First People's Hospital of Chenzhou, No. 102, Luo Jiajing, 423000, Chenzhou City, Hunan Province, China; c Department of Hepatobiliary Surgery, The First People's Hospital of Chenzhou, No. 102, Luo Jiajing, 423000, Chenzhou City, Hunan Province, China

**Keywords:** HCC, circGDI2, Glycolysis, m^6^A modification, IGF2BP2

## Abstract

**Background:**

Hepatocellular carcinoma (HCC) is a highly aggressive malignancy, and metabolic reprogramming, particularly glycolysis, plays a crucial role in its progression. Circular RNAs (circRNAs) and N6-methyladenosine (m^6^A) modifications have emerged as key regulators in HCC, but the role of circGDI2 and its underlying mechanisms remain unclear.

**Objective:**

This study aimed to investigate the functional role and mechanism of circGDI2 in HCC proliferation and glycolysis.

**Methods:**

The expression of circGDI2 was detected by RT-qPCR. RNase R treatment was used to verify the stability of circGDI2. Functional assays, including CCK-8, glycolysis analysis (glucose consumption, lactate production), and xenograft model, were performed to assess proliferation and glycolysis. Bioinformatics prediction, MeRIP, and luciferase reporter assays were used to explore the interaction between circGDI2, IGF2BP2, and PKM2.

**Results:**

CircGDI2 was highly expressed in HCC tissues and cells, and exhibited cytoplasm localization. Silencing circGDI2 inhibited Li-7 and Huh-7 cell proliferation and glycolysis, downregulated PKM2, and suppressed tumor growth. Mechanistically, circGDI2 regulated PKM2 through the m^6^A “reader” IGF2BP2, and its overexpression partially rescued the inhibitory effects of circGDI2 knockdown. Furthermore, FTO-mediated m^6^A modification enhanced circGDI2 stability and expression. Silencing FTO inhibited HCC cell proliferation, glycolysis, and tumor growth, and decreased the levels of circGDI2, IGF2BP2, and PKM2.

**Conclusions:**

FTO-mediated m^6^A modification of circGDI2 promotes HCC proliferation and glycolysis via the IGF2BP2/PKM2 axis, suggesting circGDI2 as a potential therapeutic target for HCC.

## Graphical abstract

Elevated FTO expression in hepatocellular carcinoma cells removes m6A modification on circRNA GDI2, circRNA GDI2 expression is elevated and binds to IGF2BP2 to promote IGF2BP2 expression, and highly expressed IGF2BP2 promotes the expression of PKM2 thereby enhancing the level of glycolysis in the cancer cells, and promoting the malignant process of hepatocellular carcinoma.

**Figure:**
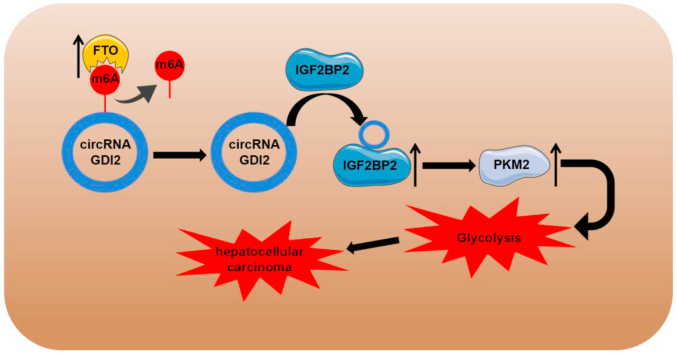

## 1 Introduction

Hepatocellular carcinoma (HCC) stands as one of the most prevalent cancers [1]. The morbidity rate of HCC ranks sixth globally and its fatality rate ranks third [1]. At present, there are many treatment methods such as surgical resection, liver transplantation, ablation, systemic chemotherapy with chemotherapy drugs, neoadjuvant immunotherapy, etc., which provide multiple options for HCC patients and significantly prolong their survival period [2,3]. Notably, most HCC patients exhibit no apparent clinical symptoms during the early stages. Consequently, this aggressive disease often goes undetected until it reaches advanced stages, thereby forfeiting the opportunity for radical surgery [4]. The characteristics of HCC being prone to recurrence and metastasis result in a dismal five-year survival rate below 20 % [5]. There is an urgent need to better understand the molecular mechanisms driving HCC progression and to identify novel therapeutic targets for more effective treatments.

To meet the substantial material and energy demands required for rapid growth and proliferation, tumor cells frequently undergo metabolic reprogramming [6]. Aerobic glycolysis, a hallmark of altered energy metabolism in tumor cells, is a critical regulator of tumor progression [7,8]. The glycolytic intermediates in the aerobic glycolysis process can synthesize nucleotides, amino acids and lipids, and simultaneously produce adenosine triphosphate (ATP) [9]. Studies have shown that glycolysis facilitates various processes in HCC cells, including proliferation, invasion, immune evasion, metastasis, angiogenesis, and drug resistance [[10], [11], [12]]. There are mainly three rate-limiting enzymes in glycolysis, namely hexokinase 2 (HK2), phosphofructokinase 1 (PFK-1), and pyruvate kinases type M2 (PKM2) [13]. Research has demonstrated a strong association between PKM2 and tumorigenesis and metastasis, with PKM2 knockdown significantly inhibiting tumor cell migration and invasion [14,15]. Therefore, targeted regulation of PKM2 in glycolysis, holds promise as a potential therapeutic strategy for HCC.

Circular RNAs (circRNAs) represent a unique category of single-stranded, covalently closed non-coding RNA molecules that exhibit tissue-specific expression patterns during development [16]. CircRNAs have emerged as critical regulators in the occurrence and prognosis of various diseases, such as the development of tumors, nerve cells and autoimmune responses [[17], [18], [19]]. Notably, research has identified circRNAs as promising molecular markers for HCC diagnosis and potential therapeutic intervention targets [20,21]. Currently, most studies focus on how circRNAs act as miRNA sponges to upregulate or downregulate downstream mRNAs [22,23]. However, circRNAs exert biological effects through intricate interactions with RNA-binding proteins (RBPs), forming a crucial aspect of their regulatory mechanisms [16,24]. CircGDI2, also designated as circ_0005379, originates from exons 2 through 5 of the GDI2 gene, spanning chromosome chr10:5827104–5842668, and yields a mature spliced transcript of 674 nucleotides. Emerging evidence highlights its elevated expression in HCC, suggesting a potential role in HCC biology [25]. However, no study has defined its regulatory role in HCC.

Epigenetic modifications of RNA play an important regulatory role in various pathophysiological processes [26]. N6-methyladenosine (m^6^A) is the predominant internal chemical modification in eukaryotic RNA [27]. Studies have confirmed that abnormal m^6^A modification regulates tumor progression [27]. Furthermore, the differential expression of circRNA in diseases may be affected by abnormal m^6^A modification [28]. For instance, Xue et al. discovered that m^6^A modification promoted HCC by stabilizing circSTX6 [28]. Nevertheless, the precise involvement of m6A modification in HCC glycolysis and its potential influence on circGDI2 regulation remains poorly understood.

Our findings indicated that silencing circGDI2 inhibited HCC cell proliferation and glycolysis and suppressed PKM2 level, and this inhibitory effect was mediated by IGF2BP2. Additionally, FTO-driven m^6^A modification of circGDI2 increased its expression, whereas silencing FTO suppressed HCC tumor growth and decreased circGDI2, IGF2BP2, and PKM2. This study seeks to elucidate how circGDI2 modulates glycolysis to drive HCC progression, offering valuable insights for future diagnostic and therapeutic strategies in HCC management.

## 2 Material and methods

### 2.1 Tissues sample collection

HCC tissues and adjacent healthy tissues were obtained from 30 surgical patients at The First People's Hospital of Chenzhou from October 2023 to October 2024. These individuals had not undergone any prior chemotherapy, radiation treatment, or immunotherapy before their procedures. All tissues were pathologically diagnosed and the patients filled out the informed consent form prior to operation. This research was approved by the Ethics Committee of The First People's Hospital of Chenzhou (Approval Number 2023124).

### 2.2 Cell culture

THLE-2, Li-7, Huh-7, Hep G2 and Hep 3B2.1-7 were all sourced from the National Collection of Authenticated Cell Cultures (Shanghai, China). THLE-2 cells were cultivated in BEGM culture medium (Cat. No. cc-3170, Lonza, Switzerland) containing 5 ng/mL EGF (Cat. No. 62229-50-9, Sigma, USA), 70 ng/mL of phosphoethanolamine (Cat. No. 1071-23-4, Sigma, USA) and 10 % fetal bovine serum (Cat. No. 10091148, FBS, Gibco, USA). Li-7, Huh-7, Hep G2 and Hep 3B2.1-7 cells were respectively cultivated in RPMI 1640 (Cat. No. 11875093, Gibco, USA), DMEM, MEM, and MEM containing 10 % FBS. All cells were incubated in a constant temperature incubator at 37 °C and 5 % CO_2_.

### 2.3 Cell transfection and treatment

For overexpression of IGF2BP2 and FTO, the cDNA sequences of IGF2BP2 and FTO were cloned into pcDNA vector (named OE-IGF2BP2 and OE-FTO), with an empty vector acting as the OE-NC. To knock down circGDI2, WTAP, YTHDF1, ALKBH5, FTO, METTL3, and METTL14 expression, short hairpin RNA (shRNA) against circGDI2, WTAP, YTHDF1, ALKBH5, FTO, METTL3, METTL14, and negative control (named sh-circGDI2, sh-WTAP, sh-YTHDF1, sh-ALKBH5, sh-FTO, sh-METTL3, sh-METTL14, and sh-NC) were synthesized by Genepharma. The sequences are listed in Table 1. Li-7 and Huh-7 cells were cultured to a density of 60 %, transfection of 2.5 μg plasmids was performed using Lipofectamine 2000 (Cat. No. 11668500, Invitrogen, USA) for 48 h.

**Table 1:** Sequences of shRNA for cell transfection.

Target	Sequence (5′-3′)
sh-circGDI2	GATTTGCAAGGAATGTATCCTTT
sh-WTAP	GGTTCGATTGAGTGAAACAGA
sh-YTHDF1	GAAGGATACAGTTCATGACAA
sh-ALKBH5	GTGGATATGCTGCTGATGAAA
sh-FTO	GGACAAGATGAAGTGGACATT
sh-METTL3	GTTAGAGAAGAAGTTGCTACA
sh-METTL14	GCCGTGTTAAATAGCAAAGAT
sh-NC	UUCUCCGAACGUGUCACGUTT

In order to identify the stability of circRNA, the cells were separated into Mock and RNase R groups. The total RNA in the Mock group did not receive any treatment, while the total RNA samples in the RNase R group reacted with 3 U/μg RNase R (Cat. No. RNR07250, Sigma., USA) for 20 min. The linear and circular forms of circGDI2 were quantified through RT-qPCR.

### 2.4 Fluorescence in situ hybridization (FISH) assay

For subcellular localization studies, FISH was conducted using a standardized kit (Cat. No. C10910, RiboBio, China),. The circGDI2 probe sequences are provided in Table 2. Following a PBS prehybridization step, the fixed cells were incubated overnight with the fluorescent probe. Nuclei were subsequently counterstained with DAPI (Cat. No. C1006, Beyotime, China). Fluorescence imaging was carried out using a fluorescence microscope (Nikon, Japan).

**Table 2:** Sequences of circGDI2 probe for FISH assay.

Gene	Sense (5′-3′)	Antisense (5′-3′)
circGDI2	AGAACTGCCCCAAGGATTTGCAAGGAATGTATCCTGTCAGGTATAATG	CATTATACCTGACAGGATACATTCCTTGCAAATCCTTGGGGCAGTTCT

### 2.5 Cell counting Kit-8 (CCK-8)

Cell proliferation was detected using the CCK-8 reagent (Cat. No. C0037, Beyotime, China). Briefly, Li-7 and Huh-7 cells were seeded into 96-well plates at a density of 5 × 10^3^ cells/well and cultured overnight at 37 °C with 5 % CO_2_. 10 μL of CCK-8 reagent was added to each well, and the plates were incubated at 37 °C for 2 h at 24, 48, and 72 h post-transfection. The optical density value at 450 nm was determined using a microplate reader (Bio-Rad, USA).

### 2.6 Glucose consumption detection

The glucose consumption was quantified using the Glucose Assay Kit with O-toluidine (Cat. No. S0201S, Beyotime, China). Following lysis of Li-7 and Huh-7 cells with appropriate buffer, the samples were centrifuged at 12,000×*g* for 5 min to isolate the supernatant. 5 μL supernatant was combined with 185 μL of the assay reagent and heated at 95 °C on a PCR instrument for 8 min. After cooling to 4 °C, absorbance readings were taken at 630 nm using a microplate reader (Bio-Rad, USA).

### 2.7 Lactate production detection

Lactate production was assessed with the Lactate Assay Kit (Cat. No. MAK570, Sigma, USA). The cell supernatant was incubated with 50 μL of reaction mixture for 30 min before measuring absorbance at 570 nm on a Bio-Rad microplate reader.

### 2.8 Methylated RNA immunoprecipitation (MeRIP)

The total RNA was isolated and RNAiso Plus (Cat. No. 9108Q, Takara) was used for total RNA extraction, followed by DNase treatment to eliminate genomic DNA contamination. The m^6^A RNA enrichment kit (Cat. No. P-9018, EpigenTek, USA) was used for MeRIP. The procedure involved immunoprecipitation with beads attached to m^6^A-specific antibodies to capture methylated RNA fragments. Following precipitation, targeted RNA sequences flanking m^6^A sites were enzymatically cleaved using a specialized lyase mixture. The enriched RNA was subsequently purified and collected for analysis. Post-immunoprecipitation, quantitative PCR was performed to assess differential methylation patterns in the target genes.

### 2.9 Dual-luciferase reporter assay

Wild-type (WT) and mutant (Mut) circGDI2 sequences containing the FTO binding site were respectively inserted into the pGL3 vector. Following plasmid transfection into cells cultured in 24-well plates, cell activity was measured after 48 h using the Dual-Luciferase Reporter Assay System (Cat. No. E1960, Promega, USA).

### 2.10 Tumor xenograft model

The BALB/c nude mice (6–8 weeks old) from Jiangsu Huachuang Sino were utilized for xenograft studies under protocols approved by the Ethics Committee of The First People's Hospital of Chenzhou (Approval Number 2023124). Mice were acclimated to the SPF-grade animal facility for 1 week before the experiment, with free access to sterilized standard chow and autoclaved water. The feeding cycle maintained a 12-h light/dark cycle at 22 ± 2 °C and 50 ± 5 % humidity, with bedding changed daily to ensure cleanliness.

To explore the role of circGDI2 and IGF2BP2 in HCC, mice were randomized into four cohorts (N = 3): sh-NC, sh-circGDI2, sh-circGDI2+OE-NC, and sh-circGDI2+OE-IGF2BP2. To explore the role of FTO in HCC, mice were allocated to sh-NC or sh-FTO groups.

Huh-7 cells (2 × 10^6^ cells) with stably transfected sh-circGDI2, OE-IGF2BP2, sh-FTO, or controls were subcutaneously injected into the right flank. Tumor volume was evaluated at three-day intervals. Three weeks later, the mice were euthanized via intraperitoneal injection of 200 mg/kg sodium pentobarbital (3 %), followed by tumor extraction.

### 2.11 Hematoxylin and eosin (HE) staining

The isolated nude mouse transplanted tumors underwent standard fixation, paraffin embedding, and sectioning at 5 μm. The slices were dewaxed in xylene solution, dehydrated with gradient ethanol, stained successively with hematoxylin solution (Cat. No. Y269827, Beyotime, China), and eosin. The images were observed under an optical microscope (Nikon, Japan).

### 2.12 Terminal deoxynucleotidyl transferase mediated dUTP nick-end labeling (tunel)

A TUNEL Cell Apoptosis Detection Kit (Cat. No. C1098, Beyotime, China) was used to detect apoptotic cells in tumour tissues, with the process being carried out according to the manufacturer's instructions. Colour development was achieved using DAB, followed by hematoxylin restaining, differentiation, dehydration and mounting. The stained sections were then examined using a Nikon microscope.

### 2.13 Immunohistochemical (IHC) staining

The sections were antigen-repaired with sodium citrate-EDTA solution (Cat. No. P0086, Beyotime, China). Endogenous peroxidase activity was blocked, followed by overnight incubation with primary antibodies at 4 °C. After incubation with secondary antibody for 30 min, DAB was added for color development and counterstained with hematoxylin. After dehydration and sealing, it was examined under an optical microscope (Nikon, Japan). The antibody information is shown in Table 3.

**Table 3:** The antibody information for IHC and Western blot.

Antibody	Experiment	Host	Diluted multiples	Company	Catalog No.
IGF2BP2	Western blot	Rabbit	1:5000	Proteintech	11601-1-AP
PKM2	Western blot	Rabbit	1:5000	Proteintech	15822-1-AP
β-actin	Western blot	Rabbit	1:9000	Proteintech	20536-1-AP
HRP-conjugated Goat Anti-Rabbit IgG (H + L)	Western blot	Goat	1:10000	Proteintech	SA00001-2
Ki67	IHC	Rabbit	1:500	Proteintech	84432-1-RR
IGF2BP2	IHC	Rabbit	1:500	Proteintech	11601-1-AP
PKM2	IHC	Rabbit	1:500	Proteintech	15822-1-AP
HRP-Goat Anti-Rabbit Secondary Antibody (H + L)	IHC	Rabbit	1:500	Proteintech	RGAR011

### 2.14 RT-qPCR

Total RNA of tissues and cells was extracted using TRIzol® reagent (Cat. No. 15596018CN, Invitrogen, USA). The first strand cDNA was synthesized using the Prime-Script RT kit (Cat. No. RR037Q, Takara, Japan). Gene expression analysis was conducted via RT-qPCR with SYBR Premix Ex *Taq*II (Cat. No. RR390L, Takara, USA). Relative quantification was determined by 2^−ΔΔCT^ and standardized by β-actin or U6. Primer sequences are detailed in Table 4.

**Table 4:** The primer sequences for RT-qPCR.

Gene	Forward Primer (5′-3′)	Reverse Primer (5′-3′)
CircGDI2	CATGGTTCCCGCTACTGTGT	TAGGCCTCGAATCCACCGAT
GDI2	TCAATGGGGAGAGGAAGAGA	CTGCTTCAGTGGAAGGAACC
HIF-1α	GCTTTAACTTTGCTGGCCCC	TTTTCGTTGGGTGAGGGGAG
HK1	TCATCACACTGGGCACTGAC	CGGAATACTCGGACCAGACG
PKM2	GGAAGTGGGCAGCAAGATCT	GCTCGACCCCAAACTTCAGA
PFKM	CCGTGGTTCTCGTCTCAACA	CGGGTGTCATATCCCAGACG
HK2	GATTGCCTCGCATCTGCTTG	GCTCCAAGCCCTTTCTCCAT
GLUT1	ACTCATGACCATCGCGCTAG	GGACCCTGGCTGAAGAGTTC
PFKP	GCATGGGTATCTACGTGGGG	CTCTGCGATGTTTGAGCCTC
LDHA	ACCGTGTTATTGGAAGCGGT	CTCCATGTTCCCCAAGGACC
IGF2BP2	CAGATTGATTTCCCGCTGCG	GCATGCTTCAGAAGTCCCCT
β-actin	CATGTACGTTGCTATCCAGGC	CTCCTTAATGTCACGCACGAT
U6	CTCGCTTCGGCAGCACA	AACGCTTCACGAATTTGCGT

### 2.15 Western blot

For protein analysis, tissue and cellular proteins were extracted using RIPA (Cat. No. R0278, Sigma, USA) and a 1 % protease inhibitor mixture (Cat. No. P8340, Sigma-Aldrich, USA). Samples were resolved on 10 % polyacrylamide gels via electrophoresis, followed by transfer to polyvinylidene fluoride membranes. After blocking with 5 % non-fat milk, membranes were probed with primary antibodies overnight at 4 °C overnight. Thoroughly wash the membrane, transfer it to the diluted secondary antibody solution and incubate at 37 °C for 2 h. Protein detection was achieved using enhanced chemiluminescence reagents (Cat. No. 32134, Thermo Fisher Scientific, USA). The antibody information is shown in Table 3.

### 2.16 Bioinformatics analysis

The Circular RNA Interactome database (https://circinteractome.nia.nih.gov/) was used to predict potential binding sites between circGDI2 and IGF2BP2.

Starbase v2.0 (http://starbase.sysu.edu.cn/https://rnasysu.com/encori/rbpClipRNA.php?source=mRNA) was used to analyze potential binding motifs between IGF2BP2 protein and PKM2 mRNA.

The SRAMP online tool (https://www.cuilab.cn/m6asiteapp/old) was used to predict m6A modification sites in circGDI2.

### 2.17 Statistical analysis

Statistical evaluations were carried out in GraphPad Prism 6.0, with data presented as mean ± SD. Between-group comparisons were assessed using independent *t*-tests, while multi-group differences were analyzed via one-way ANOVA with Tukey's post hoc test. *P* value < 0.05 was deemed statistically significant.

## 3 Results

### 3.1 circGDI2 is highly expressed in HCC tissues and cell lines

**Fig. 1:**
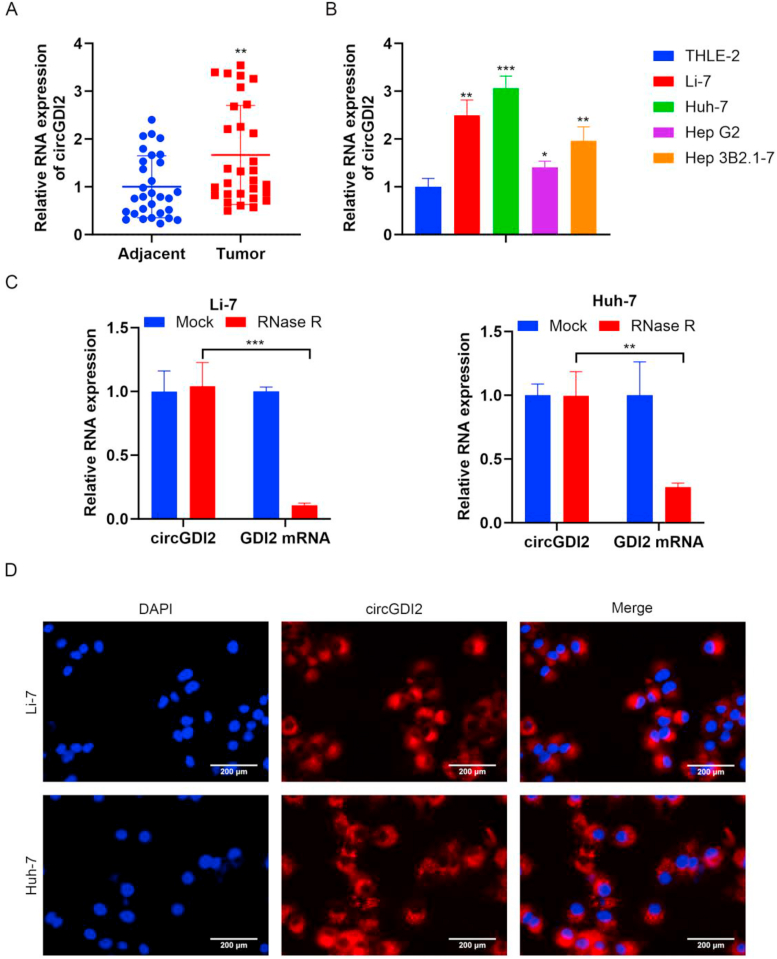
circGDI2 is highly expressed in HCC tissues and cell lines. **(A)** The expression levels of circGDI2 in HCC tissues and adjacent normal tissues were detected by RT-qPCR. **(B)** The expression levels of circGDI2 in normal cell line THLE-2 and different human HCC cell lines (Li-7, Huh-7, Hep G2 and Hep 3B2.1-7) were detected by RT-qPCR. **(C)** Li-7 and Huh-7 cells were treated with RNase R to induce linear RNA degradation, and RT-qPCR was used to detect the linear and circular parts of circGDI2. **(D)** FISH was performed to visualize the subcellular localization of circGDI2 in Li-7 and Huh-7 cells (scale bar = 200 μm). ∗*P* < 0.05, ∗∗*P* < 0.01, ∗∗∗*P* < 0.001, *vs.* the Adjacent group, the THLE-2 group, or the Mock group.

The expression levels of circGDI2 in HCC tissues and cells were detected by RT-qPCR. As indicated by the results, the expression level of circGDI2 in HCC tissues was higher than that in adjacent normal tissues (Fig. 1A). Consistently, circGDI2 level in different human HCC cell lines (Li-7, Huh-7, Hep G2 and Hep 3B2.1-7) was increased compared with the normal cell line THLE-2 (Fig. 1B). The expression was the highest in Li-7 and Huh-7 cells (Fig. 1B). Therefore, these two cell lines were selected for subsequent experimental verification.

Subsequently, Li-7 and Huh-7 cells were treated with RNase R to induce linear RNA degradation. The data demonstrated that compared with the Mock group, the linear form of circGDI2 expression in Li-7 and Huh-7 was decreased after RNase R treatment (Fig. 1C). However, the expression level of the circular form of circGDI2 remained unchanged, indicating that RNase R had no effect on the stability of circular RNA (Fig. 1C). Additionally, FISH confirmed the cellular distribution of circGDI2, revealing its predominant cytoplasm localization in HCC cells (Fig. 1D). The above results indicate that circGDI2 is highly expressed in HCC tissues and cell lines, which is a circular RNA that is stably expressed in the nuclei of HCC cells.

### 3.2 Silencing circGDI2 inhibits proliferation and glycolysis in HCC cells

**Fig. 2:**
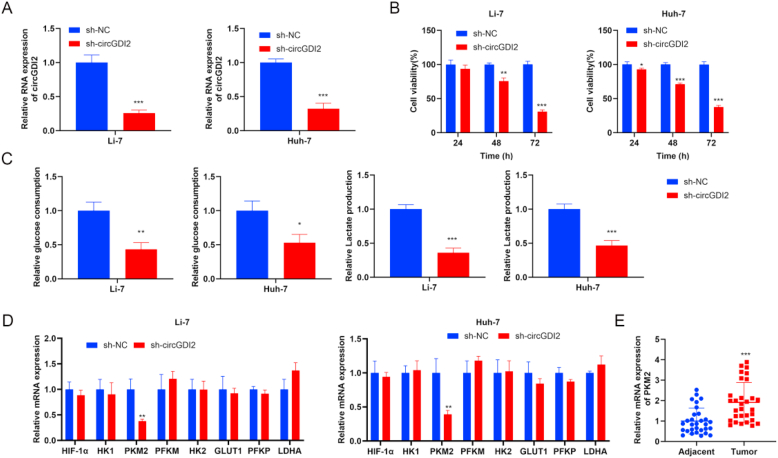
Silencing circGDI2 inhibits proliferation and glycolysis in HCC cells To explore the effect of circGDI2 on proliferation and glycolysis in HCC cells, sh-circGDI2 was transfected into Li-7 and Huh-7 cells. **(A)** The transfection efficiency was confirmed by RT-qPCR. **(B)** CCK-8 assay was used to assess cell proliferation. **(C)** Glucose consumption level and lactate production were detected using the Glucose Assay Kit with O-toluidine and Lactate Assay Kit. **(D)** RT-qPCR was used to analyze the expression changes of key glycolysis-related genes (HIF-1α. HK1, PKM2, PFKM, HK2, GLUT1, PFKP, LDHA) in Li-7 and Huh-7 cell lines after circGDI2 knockdown. **(E)** The expression level of PKM2 was examined in clinical HCC tissues and adjacent normal tissues using RT-qPCR. ∗*P* < 0.05, ∗∗*P* < 0.01, ∗∗∗*P* < 0.001, *vs.* the sh-NC group, or the Adjacent group.

To explore the effect of circGDI2 on proliferation and glycolysis in HCC cells, sh-circGDI2 was transfected into Li-7 and Huh-7 cells. Compared with the sh-NC group, circGDI2 level was decreased in the sh-circGDI2 group (Fig. 2A). CCK-8 assay showed that silencing circGDI2 inhibited cell proliferation in Li-7 and Huh-7 cells (Fig. 2B). Glycolysis is characterized by increased consumption of glucose and production of lactate. The data suggested that silencing circGDI2 decreased glucose consumption level and lactate production of Li-7 and Huh-7 cells (Fig. 2C). Then, RT-qPCR was used to analyze the expression changes of key glycolysis-related genes (HIF-1α. HK1, PKM2, PFKM, HK2, GLUT1, PFKP, LDHA) in Li-7 and Huh-7 cell lines after circGDI2 knockdown. CircGDI2 knockdown notably down-regulated PKM2 level in Li-7 and Huh-7 cells (Fig. 2D). The expression level of PKM2, a key glycolytic enzyme, was examined in clinical HCC tissues and adjacent normal tissues using RT-qPCR. The result showed that the expression of PKM2 was significantly higher in HCC tissues compared to adjacent normal tissues (Fig. 2E). These findings suggest that silencing circGDI2 plays an important role in promoting the proliferation and glycolysis of HCC cells.

### 3.3 Silencing circGDI2 inhibits proliferation and glycolysis and PKM2 expression through IGF2BP2 in HCC cells

**Fig. 3:**
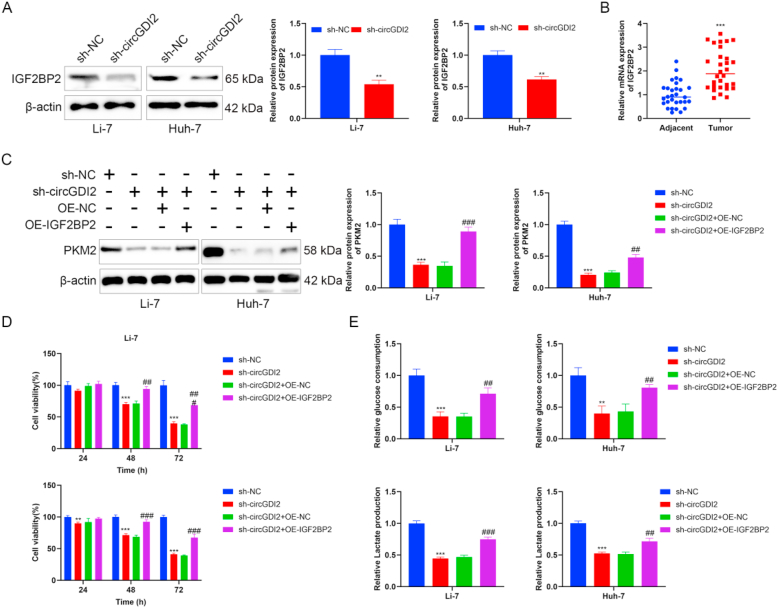
Silencing circGDI2 inhibits proliferation and glycolysis and PKM2 expression through IGF2BP2 in HCC cells. **(A)** Western blot was used to analyze IGF2BP2 expression in Li-7 and Huh-7 cell lines after circGDI2 knockdown. **(B)** The expression level of IGF2BP2 was examined in clinical HCC tissues and adjacent normal tissues using RT-qPCR. To clarify if circGDI2 regulated HCC cell proliferation and glycolysis through IGF2BP2, sh-circGDI2 and OE-IGF2BP2 were co-transfected into Li-7 and Huh-7 cells. **(C)** Western blot was used to assess the effect of IGF2BP2 on the expression of PKM2. **(D)** CCK-8 assay was used to assess cell proliferation. **(E)** Glucose consumption level and lactate production were detected using the Glucose Assay Kit with O-toluidine and Lactate Assay Kit. ∗*P* < 0.05, ∗∗*P* < 0.01, ∗∗∗*P* < 0.001, *vs.* the sh-NC group, or the Adjacent group. ^#^*P* < 0.05, ^##^*P* < 0.01, ^###^*P* < 0.001, *vs.* the sh-circGDI2+OE-NC group.

Accumulating evidence supports that the interaction between circRNA and RBPs is an important part for circRNA to achieve biological functions [16,24]. Previous studies have shown that IGF2BP2, an m^6^A “reader”, participates in the glycolysis process of HCC [29,30]. The bioinformatics tool (Circular RNA Interactome) predicted that there were potential sites for binding to IGF2BP2 on the circGDI2 sequence fragment (Fig. S1A). The bioinformatics tool (Starbase v2.0) predicted the IGF2BP2 protein structure contained possible sites for interaction with PKM2 mRNA (Fig. S1B). The prediction results suggest that IGF2BP2 is an important factor mediating the regulation of PKM2 by circGDI2. Western blot showed that silencing circGDI2 significantly reduced IGF2BP2 expression (Fig. 3A). Besides, RT-qPCR revealed significantly elevated IGF2BP2 expression in HCC tissues (Fig. 3B).

To clarify if circGDI2 regulated HCC cell proliferation and glycolysis through IGF2BP2, sh-circGDI2 and OE-IGF2BP2 were co-transfected into Li-7 and Huh-7 cells. Western blot showed that silencing circGDI2 reduced PKM2 level, which was partially reversed by IGF2BP2 overexpression (Fig. 3C). CCK-8 showed that the Li-7 and Huh-7 cell proliferation ability of the sh-circGDI2 group was weaker than the sh-NC group, and the proliferation ability of the sh-circGDI2+OE-IGF2BP group was higher than the sh-circGDI2 group (Fig. 3D). Besides, the inhibitory influence of sh-circGDI2 on glucose consumption level and lactate production was partially rescued by IGF2BP2 overexpression (Fig. 3E). Taken together, these results confirm the role of silencing circGDI2 on HCC cell proliferation and glycolysis is mediated by IGF2BP2.

### 3.4 Silencing circGDI2 inhibits HCC tumor growth and PKM2 expression through IGF2BP2

**Fig. 4:**
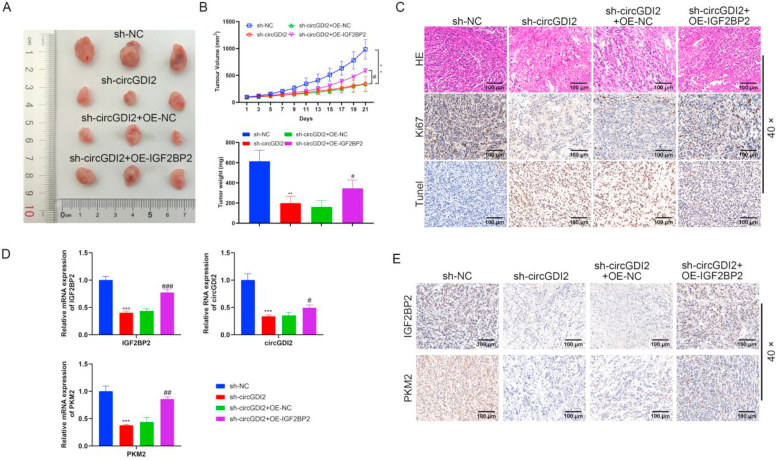
Silencing circGDI2 inhibits HCC tumor growth and PKM2 expression through IGF2BP2. To verify the effect of circGDI2 and IGF2BP2 on HCC tumor growth, a xenograft mouse model was constructed. **(A)** Pictures of the isolated tumors of the indicated group. **(B)** The tumor volume and weight were recorded. **(C)** HE staining, Tunel and Ki-67 staining were performed to observe the histological characteristics and cell proliferation in the tumor tissues (scale bar = 100 μm). **(D)** RT-qPCR was used to detect circGDI2, IGF2BP2 and PKM2 levels. **(E)** IHC was used to detect IGF2BP2 and PKM2 levels (scale bar = 100 μm). ∗*P* < 0.05, ∗∗*P* < 0.01, ∗∗∗*P* < 0.001, *vs.* the sh-NC group. ^#^*P* < 0.05, ^##^*P* < 0.01, ^###^*P* < 0.001, *vs.* the sh-circGDI2+OE-NC group.

To assess the impact of circGDI2 and IGF2BP2 on HCC tumor tumorigenesis, a xenograft mouse model was constructed. The results elucidated that silencing circGDI2 significantly suppressed the tumor weight and volume, and overexpressing IGF2BP2 partially reversed the inhibitory influence of circGDI2 knockdown (Fig. 4A and B). HE staining suggested that the tumor cells in the sh-NC group grew well, were closely arranged, had a large cell volume, and the nuclear staining was clear (Fig. 4C). The tumor cells in the sh-circGDI2 group, necrotic areas emerged, with sparse cell arrangement and incomplete cell morphology (Fig. 4C). CircGDI2 knockdown led to disrupted tumor tissue, and overexpressing IGF2BP2 partially reversed these effects (Fig. 4C). IHC suggested that silencing circGDI2 reduced the number of Ki-67 positive cells, indicating that silencing circGDI2 inhibited the cell proliferation of transplanted tumors (Fig. 4C). Meanwhile, IGF2BP2 overexpression further increased the number of Ki-67 positive cells, indicating that IGF2BP2 reversed the inhibitory effects of silencing circGDI2 on tumor proliferation (Fig. 4C). Tunel staining showed that inhibition of circGDI2 promoted tumor cell apoptosis, which was reversed by overexpression of IGF2BP2 (Fig. 4C). RT-qPCR showed that circGDI2 knockdown down-regulated the expressions of circGDI2, IGF2BP2 and PKM2, and IGF2BP2 overexpression enhanced IGF2BP2 and PKM2 levels (Fig. 4D). IHC also elucidated that overexpressing IGF2BP2 abolished the inhibitory roles of silencing circGDI2 on IGF2BP2 and PKM2 protein expression (Fig. 4E). These findings unravel that circGDI2 can affect HCC tumorigenesis by regulating IGF2BP2 and PKM2.

### 3.5 FTO-mediated m^6^A modification of circGDI2 modulates proliferation and glycolysis in HCC cells

**Fig. 5:**
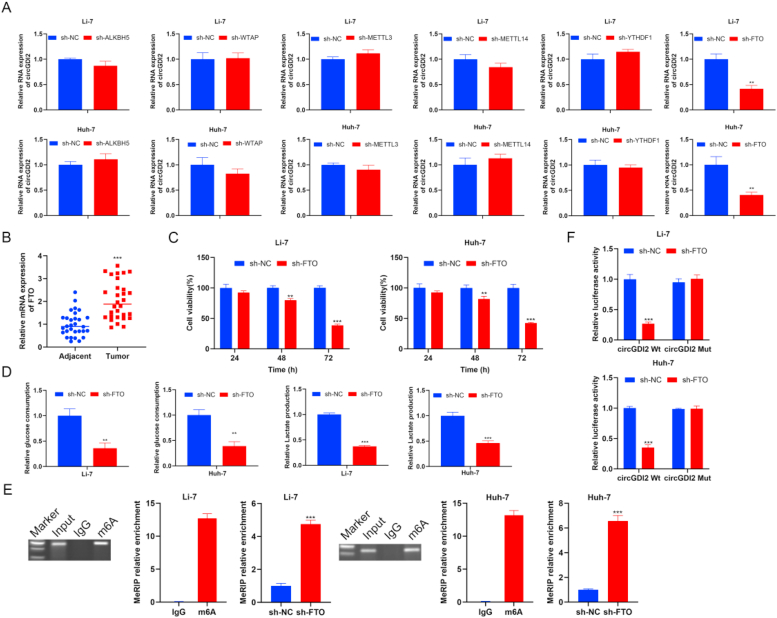
FTO-mediated m^6^A modification of circGDI2 modulates proliferation and glycolysis in HCC cells. **(A)** M^6^A methyltransferases (WTAP, METTL3, METTL14, FTO, ALKBH5 and YTHDF1) were knocked down in Li-7 and Huh-7 cells, and circGDI2 level was assessed by RT-qPCR. **(B)** The expression level of FTO was examined in clinical HCC tissues and adjacent normal tissues using RT-qPCR. To explore the effect of FTO on proliferation and glycolysis in HCC cells, sh-FTO was transfected into Li-7 and Huh-7 cells. **(C)** CCK-8 assay was used to assess cell proliferation. **(D)** Glucose consumption level and lactate production were detected using the Glucose Assay Kit with O-toluidine and Lactate Assay Kit. **(E)** MeRIP was performed to analyze the m^6^A modification status of circGDI2. **(F)** A luciferase reporter assay was used to investigate the effect of m^6^A modification on circGDI2. ∗*P* < 0.05, ∗∗*P* < 0.01, ∗∗∗*P* < 0.001, *vs.* the sh-NC group, the Adjacent group, or the OE-NC group.

M^6^A modification exerts a crucial influence on posttranscriptional regulation and the generation of circRNAs [31,32]. Through the prediction of the SRAMP online website, it was found that there were m^6^A modification sites in circGDI2 (Fig. S1C). Then, m^6^A methyltransferases (WTAP, METTL3, METTL14, FTO, ALKBH5 and YTHDF1) were knocked down in Li-7 and Huh-7 cells, and circGDI2 level was assessed. RT-qPCR clarified that sh-WATP, sh-WTAP, sh-YTHDF1, sh-ALKBH5, sh-FTO, sh-METTL3, sh-METTL14 showed no significant changes on circGDI2 level, and sh-FTO resulted in a significant decrease in circGDI2 expression (Fig. 5A). RT-qPCR showed that FTO level was significantly increased in tumor tissues (Fig. 5B). Silencing FTO significantly reduced Li-7 and Huh-7 cell proliferation, glucose consumption level, lactate production (Fig. 5C and D). Furthermore, MeRIP was performed to analyze the m^6^A modification status of circGDI2. Inhibition of FTO induced the m^6^A modification level of circGDI2 (Fig. 5E). Inhibition of FTO significantly decreased the luciferase activity of circGDI2 Wt, while no notable change was observed in the circGDI2 Mut (Fig. 5F). Above all, FTO promotes the expression of circGDI2 through m^6^A modification at specific sites.

### 3.6 Silencing FTO inhibits HCC tumor growth and decreases circRNA, IGF2BP2 and PKM2 levels

**Fig. 6:**
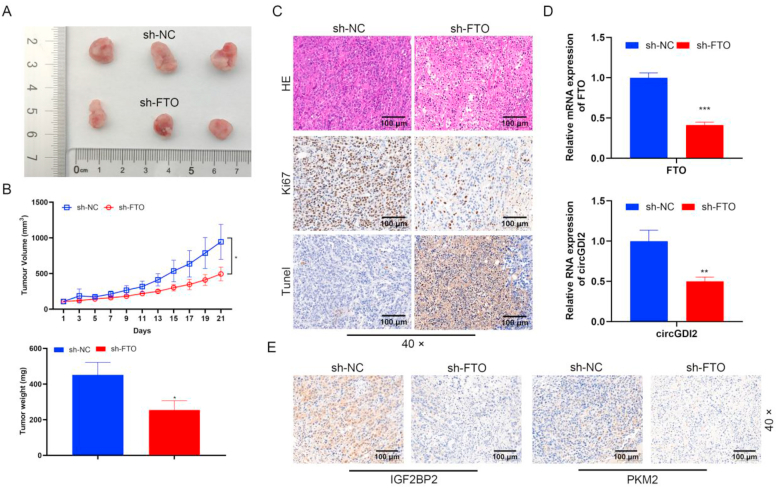
Silencing FTO inhibits HCC tumor growth and decreases circRNA, IGF2BP2 and PKM2 levels. To investigate the biological role of FTO on HCC tumor growth, the xenograft tumor models of HCC cells in the sh-NC and sh-FTO groups were established. **(A)** Pictures of the isolated tumors of the indicated group. **(B)** The tumor volume and weight were recorded. **(C)** HE staining, Ki-67 staining and Tunel stainning were performed to observe the histological characteristics and cell proliferation in the tumor tissues (scale bar = 100 μm). **(D)** RT-qPCR was used to detect circGDI2 and FTO levels. **(E)** IHC was used to detect IGF2BP2 and PKM2 levels (scale bar = 100 μm). ∗*P* < 0.05, ∗∗*P* < 0.01, ∗∗∗*P* < 0.001, *vs.* the sh-NC group.

To explore the biological role of FTO on HCC tumorigenesis, the xenograft tumor models of HCC cells in sh-NC and sh-FTO groups were established. FTO knockdown significantly suppressed the tumor weight and volume (Fig. 6A and B). HE staining showed that FTO knockdown led to disrupted tumor tissue (Fig. 6C). IHC suggested that FTO knockdown reduced the number of Ki-67 positive cells (Fig. 6C), Tunel staining results indicated that interfering with FTO expression promoted the level of apoptosis in tumor tissues (Fig. 6C). Furthermore, FTO knockdown decreased circGDI2 and FTO levels (Fig. 6D). Knockdown of FTO also decreased IGF2BP2 and PKM2 protein levels (Fig. 6E). These results demonstrate the significant impact of FTO on tumor growth and the regulation of related molecules.

## 4 Discussion

CircRNAs exhibit differential expression in HCC, strongly correlating with the disease's onset, progression, and clinical outcomes [20,21]. However, the mechanisms of circRNA in HCC remain incompletely understood. Our research revealed a marked upregulation of circGDI2 in HCC. We further demonstrated that FTO-mediated m6A modification of circGDI2 drives HCC proliferation and glycolysis by modulating the IGF2BP2/PKM2 signaling pathway.

Initial validation confirmed elevated circGDI2 expression in both HCC tissues and cell lines, along with its resistance to RNase R degradation-a hallmark of its circular structure. This is consistent with previous study [25]. Han, Zhiyi et al. confirmed through the circRNA profiling experiment that circGDI2 was increased in the peripheral blood mononuclear cells of HCC patients [25]. Furthermore, circRNAs with tissue-specific expression patterns play important roles in various cancers [33]. Subsequent functional assays revealed that silencing circGDI2 inhibited HCC tumorigenesis. All these data indicated that circGDI2 acted as a novel oncogenic circular RNA in HCC progression. In oral squamous cell carcinoma (OSCC), circGDI2 was reported as a tumor suppressor [34,35]. This phenomenon may be related to the tissue-specific microenvironment, differences in molecular interaction networks, and epigenetic regulation. For example, OSCC and HCC originate from different cell types, and there are also differences in the surrounding tissue microenvironment such as extracellular matrix components, immune cell distribution, and cytokine secretion.

The metabolic pathways of cancer cells often rely more on glycolysis rather than aerobic respiration. The alteration of this cellular metabolic mode is regarded as one of the markers of cancer [9]. Therefore, clarifying the occurrence mechanism of glycolysis is of great significance for targeted regulation of glycolysis to produce anti-tumor effects. Controlling the rate-limiting enzyme in glycolysis may inhibit tumor growth. A study reported that ZEB1 enhanced glycolysis and tumorigenesis by activating the transcription of PFKM [36]. Another study suggested that GJB2 contributed to the advancement of HCC by the activation of glycolysis [37]. We found that silencing circGDI2 suppressed glucose consumption and lactate production, indicating a suppression of glycolysis. This is further supported by the downregulation of key glycolytic enzymes such as PKM2. Evidence indicates that PKM2 is overexpressed in HCC and is related to tumorigenesis and metastasis [38,39]. We also found that PKM2 level was increased in HCC tissues. All the data indicated the critical role of circGDI2 in metabolic reprogramming.

FISH analysis revealed circGDI2 was predominant cytoplasm localization, suggesting potential roles in transcriptional regulation or protein scaffolding. Previous studies have reported that circRNAs can modulate gene expression by interacting with RBPs [40,41]. For instance, circRHBDD1 interacted with IGF2BP2 to induce immune escape in gastric cancer [40]. Besides, circITGB6 was bound to IGF2BP2 to promote ovarian cancer cisplatin resistance [41].

Previous studies have shown that IGF2BP2, an m^6^A “reader”, participates in the glycolysis process of HCC [29,30]. Wu, Tao et al. suggested that IGF2BP2 contributed to the promotion of glycolysis and the elevation of stemness in HCC through the stabilization of CDC45 mRNA [29]. Mechanistically, bioinformatics and experimental validation revealed that circGDI2 interacted with IGF2BP2 to stabilize PKM2 mRNA. IGF2BP2 overexpression partially abolished the inhibitory effects of silencing circGDI2 on HCC proliferation and glycolysis, confirming this regulatory axis. The data indicate that circGDI2 may act as a scaffold, facilitating IGF2BP2-PKM2 interaction to enhance glycolytic activity.

Recent studies highlight the importance of m^6^A modification in circRNA biogenesis and function [42,43]. The role of FTO in m^6^A modification of circGDI2 is another important aspect of our study. Among various m^6^A regulators, we identified FTO (a demethylase) as a critical factor enhancing circGDI2 expression. Knockdown of FTO reduced circGDI2 level, while MeRIP and luciferase reporter assays confirmed that FTO increases circGDI2 stability via m^6^A modification. These findings align with prior studies demonstrating FTO's critical function in posttranscriptional regulation and circRNA biogenesis [44]. Importantly, silencing FTO mirrored the effects of circGDI2 knockdown, suppressing tumor growth and down-regulating IGF2BP2/PKM2, further supporting the FTO-circGDI2-IGF2BP2-PKM2 axis in HCC progression.

Notably, recent advances in ncRNA-disease prediction models have provided new insights into the clinical application of circRNAs. Advanced models developed by domestic and international scholars, such as those utilizing multi-hop graph structural modeling and integrating high-order and low-order features with weighted attention mechanisms, are capable of efficiently capturing high-level associations within biological networks and prioritizing high-confidence candidate interactions from vast amounts of data [[45], [46], [47]]. Besides, a multi-hop graph learning model based on attention mechanisms has been used to predict Budd-Chiari syndrome by integrating high-risk factors [48]. Similarly, our study identifies circGDI2 as a potential therapeutic target for HCC, and future integration of circGDI2 expression data into such predictive frameworks may improve the accuracy of HCC diagnosis and prognosis prediction.

While our investigation provides valuable insights, certain limitations remain. First, the upstream regulatory mechanisms of circGDI2 expression and m^6^A modification need to be further explored. Second, the exact mechanism by which circGDI2 recruits IGF2BP2 requires further investigation. Third, the crosstalk between circGDI2 and other non-coding RNAs or proteins in the tumor microenvironment also requires in-depth study. Understanding these mechanisms will help to develop more effective therapeutic strategies for HCC. Additionally, the clinical correlation was performed on a single-center cohort of 30 patients. Future studies should validate these findings in larger, multi-center cohorts and incorporate survival data to definitively assess the prognostic value of circGDI2. Finally, detecting circGDI2 in liquid biopsies (e.g., plasma or serum) from HCC patients and controls would be a critical next step to evaluate its potential as a non-invasive diagnostic or monitoring biomarker in a real-world clinical setting.

## 5 Conclusion

Our study reveals the regulatory role of circGDI2 in driving HCC glycolysis and tumor growth, and the involvement of m^6^A modification mediated by FTO. Targeting the circGDI2-IGF2BP2-PKM2 axis and FTO-mediated m^6^A modification may provide new therapeutic strategies for HCC. Further research should focus on understanding the complex regulatory network in HCC and developing more effective treatment methods.

## CRediT authorship contribution statement

**Shiyi Chen:** Writing – original draft, Visualization, Investigation, Data curation, Conceptualization. **Hongxiang Xia:** Resources. **Shuwei Chen:** Writing – review & editing, Supervision, Project administration, Funding acquisition.

## Consent to participate

Informed consent was obtained from all individual participants included in the study.

## Ethics statement

This study was performed in line with the principles of the Declaration of Helsinki. Approval was granted by the Ethics Committee of The First People's Hospital of Chenzhou (approval number: 2023124).

The animals research in this study was approved by Animal Experimentation Ethics Committee of The First People's Hospital of Chenzhou (approval number: 2023124).

## Data availability statement

The data that support the findings of this study are available from the corresponding author, SWC, upon reasonable request.

## Funding

This study was supported by the 10.13039/501100004735Natural Science Foundation of Hunan Province (No. 2023JJ50367).

## Declaration of competing interest

The authors declare that they have no known competing financial interests or personal relationships that could have appeared to influence the work reported in this paper.
